# A case-study in the clinical epidemiology of psoriatic arthritis: multistate models and causal arguments

**DOI:** 10.1111/j.1467-9876.2011.00767.x

**Published:** 2011-11

**Authors:** Aidan G O'Keeffe, Brian D M Tom, Vernon T Farewell

**Affiliations:** Medical Research Council Biostatistics UnitCambridge, UK

**Keywords:** Bradford Hill criteria, Causality, Composable Markov process, Damage, Disease activity, Dynamic modelling, Granger causality and non-causality, Interval censoring, Local dependence and independence, Multistate models, Psoriatic arthritis, Random effect, Robust information sandwich estimator, Temporality

## Abstract

In psoriatic arthritis, permanent joint damage characterizes disease progression and represents a major debilitating aspect of the disease. Understanding the process of joint damage will assist in the treatment and disease management of patients. Multistate models provide a means to examine patterns of disease, such as symmetric joint damage. Additionally, the link between damage and the dynamic course of disease activity (represented by joint swelling and stress pain) at both the individual joint level and otherwise can be represented within a correlated multistate model framework. Correlation is reflected through the use of random effects for progressive models and robust variance estimation for non-progressive models. Such analyses, undertaken with data from a large psoriatic arthritis cohort, are discussed and the extent to which they permit causal reasoning is considered. For this, emphasis is given to the use of the Bradford Hill criteria for causation in observational studies and the concept of local (in)dependence to capture the dynamic nature of the relationships.

## 1. Introduction

From a clinical perspective, it is generally held that better understanding of a disease process will lead to more appropriate treatment and disease management of patients. Often multistate models (Hougaard, 1999; Commenges, 1999; Andersen and Keiding, 2002; Meira-Machado *et al.*, 2009) are particularly useful for this. In this paper we illustrate their use for the study of disease progression in psoriatic arthritis.

In his 1970 seminal paper, Schweder (1970) introduced the concept of local (in)dependence between components of a composable finite Markov process. He felt that many studied phenomena can be realistically described by time continuous finite Markov processes. If, in addition, the Markov process representing the phenomenon under study could be defined to be composable (i.e. represented as a vector of distinct subprocesses, whereby no two subprocesses or components can change state ‘simultaneously’), then (in)dependences between these subprocesses can be explicitly expressed through the transition intensities of the original Markov process.

More explicitly, let **Y** be a composable finite Markov process with components *Y*_1_,…,*Y*_*p*_, which we denote as **Y**∼(*Y*_1_,…,*Y*_*p*_), and let *V*={1,…,*p*}. Here the dependence on time in the notation is left out for convenience but is implicit. A component *Y*_*k*_ is said to be locally independent of *Y*_*j*_, *j*≠*k*, given the remaining components **Y**_*V*∖{*j*,*k*}_, if and only if the transition intensities of **Y** corresponding to transitions only between states in *Y*_*k*_ are constant functions for any state in *Y*_*j*_, within a specified infinitesimal time interval. If *Y*_*k*_ is not locally independent of *Y*_*j*_, it is said to be locally dependent on *Y*_*j*_.

The above relationship is a ‘local’ property as it holds in an infinitesimal time interval. Furthermore, recall that, because **Y**∼(*Y*_1_,…,*Y*_*p*_) is composable, no two components of **Y** can change state at the same time over this infinitesimal interval and therefore the transition intensities for such simultaneous transitions in more than one component are zero. Moreover, local independence is an asymmetric relationship, i.e. it has a direction, and so, *Y*_*k*_ being locally independent of *Y*_*j*_ does not necessarily imply that *Y*_*j*_ is locally independent of *Y*_*k*_ over the same infinitesimal time interval.

This important concept of local independence was extended in Aalen (1987) to apply to more general stochastic processes that admit a Doob–Meyer decomposition, with unrelated innovations. Aalen stressed the usefulness of ‘dynamic’ models where dynamic refers to how the future relates to the past, and it is this dynamic nature of Schweder's work, rather than the Markov assumption *per se*, which he suggested is important for statistical analysis. Earlier works by Cox (1972) and Aalen *et al.* (1980) also reflect this dynamic viewpoint. More recently, Didelez (2007, 2008) introduced dynamic graphical models to describe these local dependences, which further allowed local independences to be read off. In our discussion of the progression of psoriatic arthritis disease that follows, we use Schweder's local (in)dependence concept as a means of characterizing the findings from dynamic analyses based on multistate models.

Because we focus on arthritic disease progression at the individual joint level, our analysis is based on the use of correlated multistate models. Cook *et al.* (2004) considered such an analysis for progressive processes but with a discrete multivariate random-effects distribution used to account for correlation. For a four-state progressive model, we extend this to allow the use of gamma-distributed random effects. In addition, for a three-state model with some reversible transitions, we outline an approach that is based on generalized estimating equations to account for correlation between processes.

Our reported investigations are based on observational clinical data and it is well recognized that causal relationships can never be proved with such data. However, as Weiss (1986) argued,

‘it is necessary to attempt to draw inferences of cause and effect, even from inevitably incomplete data, for the alternative is to make no inference at all, which would preclude taking preventive or therapeutic action’.

Therefore, after presenting results of our analyses, we consider the extent to which they might allow inference concerning causal relationships. In doing so, we take up the implied challenge of Aalen (Aalen *et al.* (2008), page 348) who wrote:

‘One major danger of avoiding the subject of causality in statistical education and statistical literature, is that one never gets any insight into this fascinating concept, which has such an old history in philosophy and science. The fact is that statistics plays a major role in looking for causal connections in many fields and statisticians who know next to nothing about causality as a larger concept will be far less useful than they could have been.’

After 30 years of data collection and 20 years of previous analyses, it seems particularly appropriate to take up this challenge with the psoriatic arthritis cohort data that are discussed in the next section.

## 2. Psoriatic arthritis

### 2.1. Background

Psoriatic arthritis is an inflammatory arthritis associated with psoriasis, which is usually seronegative for rheumatoid factor (Wright and Moll, 1976; Gladman, 2004). It is distinguished as an entity from the prototypal arthritis, rheumatoid arthritis (RA), because of its unique clinical features: the association with the skin disease psoriasis; equal gender frequency, as opposed to the preponderance in females in RA; the asymmetric presentation involving large joints and distal interphalangeal joints which are not commonly affected in RA; the majority (more than 85%) of psoriatic arthritis patients lack rheumatoid factor; roughly half of the patients with psoriatic arthritis have spinal involvement which is distinctly rare in RA. Moreover, in the fingers, patients with psoriatic arthritis demonstrate dactylitis, or inflammation of the whole digit, as well as enthesitis and other extra-articular features that are typical to the seronegative spondyloarthritides. Recent studies have demonstrated that psoriatic arthritis is a progressive disease, leading to joint destruction, disability and reduced quality of life (Husted *et al.*, 2001; Kane *et al.*, 2003; McHugh *et al.*, 2003; McKenna *et al.*, 2004; Queiro-Silva *et al.*, 2003; Sokoll and Helliwell, 2001).

Disease progression in psoriatic arthritis, as with RA, is often taken to be reflected in the accumulation and severity of damaged joints, evaluated either clinically or through radiographic imaging (e.g. X-rays). Clinical damage is determined by the presence of a limitation in range of movement of more than 20% of the range not related to the presence of joint effusion, the presence of joint deformities, subluxation, flail joints or ankylosis (Siannis *et al.*, 2006). The damage process is generally irreversible; therefore once a joint is damaged it will remain so; thus efforts by clinicians to prevent or slow this process are crucial in the care of patients. Disease activity, in contrast, is a reversible process, and is reflected in part by joints being described as either tender (the presence of stress pain and/or joint line tenderness) only or effused (joint swelling with or without tenderness), with the latter representing a more severe level of activity than the former. Various types of medication are available to treat the disease activity. The use of non-steroidal anti-inflammatory drugs and disease modifying anti-rheumatic drugs as first- and second-line treatments, and more recently biologics that bind to tumour necrosis factor *α*, preventing activation of its receptors, are all available in the clinician's arsenal of medications for controlling the activity of the disease. In addition, if these front-line therapies are not effective at reducing inflammation, the use of intra-articular steroids injected directly into the specific active joint(s) may also be considered.

In 1978, the Toronto Psoriatic Arthritis Clinic was established by Professor Dafna Gladman at the University of Toronto after recognizing that there was a paucity of knowledge regarding psoriatic arthritis at the time. Since then, much has been learned about the disease through research done at the Toronto Psoriatic Arthritis Clinic, which has the largest and most comprehensively studied cohort of psoriatic arthritis patients in the world. At present, the clinic includes over 1000 patients who have been closely followed up prospectively over the years. Visits to the clinic are aimed to be scheduled 6–12 months apart. At these visits, patients are evaluated in a standard way according to a defined protocol that includes a complete history, physical examination (including rheumatological assessment) and routine blood and urine tests, with X-rays also being performed biennially. The rheumatological examination includes validated and reliable determination of active and damaged joints. This longitudinal information provides a valuable resource for research.

Up to the end of 2006, which, in the main, was the period predating the introduction of biologics, longitudinal data on 790 patients were collected from the Toronto Psoriatic Arthritis Clinic. A subset of 517 patients, which is defined in Section 3, of the 790 will be used in this study to examine patterns of disease and to investigate the link between clinical damage and the dynamic course of disease activity at both the individual joint level and otherwise. Of these 517 patients, 289 (55.9%) were male and 228 (44.1%) female. The mean age at entry was 41 years and 7 months, with a standard deviation of 12 years and 7 months. The number of visits to the clinic recorded up to 2006 ranged from 2 to 47, with a median of 7. The mean gap time (i.e. the time between successive clinic visits) was 10.8 months (standard deviation 15.2 months) and the median gap time was 6.3 months.

### 2.2. Previous investigations

For both psoriatic arthritis and RA there is a strong belief among clinicians that active inflammation (i.e. persistent inflammatory synovitis) results in or causes joint damage. A number of research groups have repeatedly shown an association between disease activity and progression to damage in both psoriatic arthritis and RA, with this association consistently seen irrespectively of the measure of activity used (i.e. whether it be the number of active (tender or effused) joints, biochemical markers or some form of composite measure of disease activity) or whether damage is determined radiologically or clinically (Aletaha *et al.*, 2009; Bond *et al.*, 2007; Gladman *et al.*, 1995; Gladman and Farewell, 1999, Molenaar *et al.*, 2004; Mulherin *et al.*, 1996; Scott, 2004; Smolen *et al.*, 2009; Welsing *et al.*, 2004).

For psoriatic arthritis, it has been found that radiological damage precedes clinical damage in the majority of patients (Siannis *et al.*, 2006), and that the same predictors of disease progression are seen regardless of how damage is detected (Bond *et al.*, 2007). In a recent investigation of the link between activity and damage in psoriatic arthritis based on the Toronto Psoriatic Arthritis data (Bond *et al.*, 2007; Bond and Farewell, 2009), negative binomial regression models for the increase in the total damaged joint count between visits were fitted, with previous damage incorporated as a dynamic explanatory variable in the models to account for the within-patient correlation. Disease activity was included initially in these models both in terms of total active joint counts (effused and tender) at entry to the clinic and as time-dependent explanatory variables, with the total joint counts updated at visits to the clinic. These models found that time varying activity (both effused and tender total joint counts) was associated with the progression of radiological and clinical damage, but that activity variables at entry to the clinic were not, when in the presence of their time varying counterparts.

These results, coupled with similar results in RA, argue for a close link between activity and damage, which, additionally, is supported by the time ordering. This argument is further strengthened by the dose–response nature of the relationship between activity and damage. In our recent psoriatic arthritis investigation, differential effects of tender-only joint and effused (usually also tender) joint counts were found to some extent, with the relative rate estimate for the more severe effused joint count larger than the relative rate estimate for tender joint count. Similar findings were reported in a recent study in RA, in which a positive association was found between the level of disease activity (defined by using the simplified disease activity index) and the level of radiographic progression (defined by using the modified total Sharp score) (Aletaha *et al.*, 2009).

However, because total joint counts or other ‘global’ measures of disease activity and damage are used, the link that was found between activity and damage is essentially between the systemic activity process and the systemic damage process. If we can consider a finer level of detail and examine the link at the individual joint level then similar results would further our understanding of the relationships between activity and damage. Additionally, issues that are related to disease patterns can be investigated.

## 3. Four-state model for damage process

**Fig. 1:**
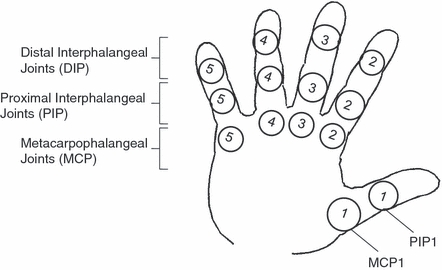
Diagram showing the 14 hand joints and their type

Clinical damage and disease activity information at the individual joint level for the hands (excluding the wrist joints) was extracted from the Toronto Psoriatic Arthritis Clinic's database. Fig. 1 shows a picture of the 14 joints in a hand. In it, three types of joint are seen: the distal interphalangeal, the proximal interphalangeal and the metacarpophalangeal. All three joint types are usually found on a digit, except for the thumb where the distal interphalangeal joint is absent. There are 28 hand joints in total.

For the purposes of this paper, we use data extracted from 517 of the 790 patients in the clinic who entered before the beginning of 2007. These 517 patients correspond to those who, at entry to the clinic, had no clinical damage in any of the hand joints on either the left or right hands and therefore information on all these patients is comparable. The clinical damage processes at the hand joint level may be observed in either of two states at a clinic visit (‘not damaged’ or ‘damaged’). However, once a hand joint enters the damaged state it remains there *ad infinitum*, i.e. the damaged state is absorbing, and all the individual joints’ damage processes are irreversible. Disease activity at a specific hand joint is defined by two joint-specific processes: a tender-only joint process and an effused joint process. Both these two activity processes are reversible. The tender joint process can move back and forth between a state of ‘no stress pain’ and a state of ‘tenderness only’. The effused joint process can move between states of ‘no swelling’ and ‘effusion’.

**Fig. 2:**
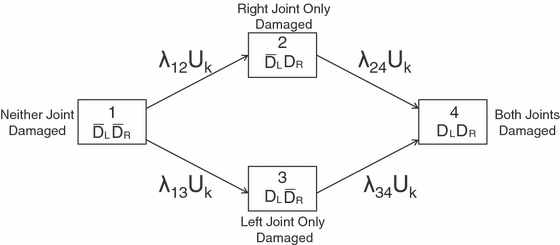
Diagram of the multistate model for damage at a joint location, with random effect

The multistate model for our first set of analyses is depicted diagrammatically in Fig. 2 and is a four-state model for damage in each of the 14 pairs of hand joints. The four states of this multistate model are defined as

- state 1, damage in neither hand, 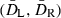,
- state 2, damage in the right hand only, 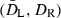,
- state 3, damage in the left hand only, 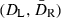, and
- state 4, damage in both hands, (*D*_L_,*D*_R_).

This is a multistate model at a specific joint location in both left and right hands. Moreover, it is composable, comprising separate damage subprocesses for the left and right hands at the specific joint location, and local dependences between the subprocesses will be reflected in relationships between the transition intensities of this multistate model. In addition, this model does not allow transitions directly from state 1 to state 4. This is a necessary constraint to ensure composability but is not very restrictive for models with transitions in continuous time. Transitions between state 2 and state 3 are not allowed since damage is irreversible. Furthermore, because the 14 multistate processes within a patient should be more similar than across patients, we introduce subject-specific random effects into the model. These act multiplicatively on the baseline transition intensities of the 517 patients. The random effects, *U*_*k*_,*k*=1,…,517, are assumed to be distributed as independent gamma random variables with unit mean and variance *θ* (see equation (6) in Appendix A), which we denote by *U*_*k*_∼gamma(1/*θ*,1/*θ*).

To be more precise, consider the continuous time multistate process for damage, 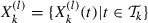, at the *l*th joint location for the *k*th patient, with state space Ω={1,…,4} as described above, and 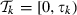 being the time interval of interest. Here the timescale is time from entry to the clinic in years. Let 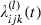 be the transition intensity function that is associated with 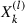 corresponding to the transition from state *i* to state *j*, denoted as *i*→*j*, where *i*≠*j* (*i*=1,2,3;*j*=2,3,4) and with transitions 1→4, 2→3 and 3→2 not permissible. Let 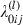 be the baseline transition intensity corresponding to this *i*→*j* transition. In addition, let 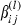 be a vector of regression parameters that are associated with the predictable explanatory variable vector process 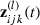. Then the transition intensity function for the *i*→*j* transition, *i*≠*j*, at the *l*th hand joint location for the *k*th patient is given by 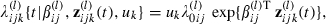
(1) where *u*_*k*_ corresponds to the subject-specific random effect. This multistate model is a generalization of Schweder's model to incorporate random effects and is similar to the model that was proposed by Cook *et al.* (2004) for clustered progressive multistate processes. Nevertheless, this model still allows us to examine local dependences through the transition intensities. Note that we have assumed that the regression parameter vector 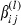 is joint location specific. However, it may be reasonable to set 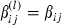 as the 14 joint location processes represent a similar disease progression phenomenon, and we do this subsequently. Also it may at times be sensible to constrain baseline intensities to be the same across some joint locations for parsimony, and we shall indicate when this has been done. Moreover, further constraints on regression parameters may be acceptable for simplification based on the analyses of the data.

### 3.1. Maximum likelihood estimation

We employ an underlying time homogeneous Markov model for panel data where patients are not under continuous follow-up and are observed only at protocol visits (Kalbfleisch and Lawless, 1985), and with baseline transition intensities modulated by the effects of explanatory variables **z**(*t*) and subject-specific random effects {*u*_*k*_} as described in model (1). Suppose that the *k*th patient makes *n*_*k*_ clinic visits at times **t**_*k*_=(*t*_*k*1_,…,*t*_*kn*_*k*__)^T^ and associated with the *l*th joint location process for this patient are an explanatory variable vector process 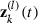 recorded at clinic visits, baseline transition intensities 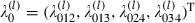 and a vector of regression parameters *β*^(*l*)^. Then the conditional probability of observing the path that is followed by the *l*th joint location process of the *k*th patient over the *n*_*k*_ clinic visits, given *u*_*k*_, is 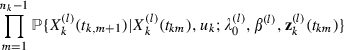 and thus the overall likelihood contribution from the *k*th patient is given by 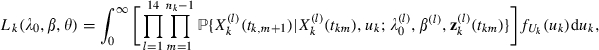 where 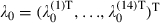, *β*=(*β*^(1)T^,…,*β*^(14)T^)^T^ and *f*_*U*_*k*__(*u*_*k*_) denotes the probability density function of the gamma(1/*θ*,1/*θ*) distribution. (The integration over the random-effect distribution can be carried out in R (R Development Core Team, 2009) by using the command.) Hence the likelihood function across all *N* individuals is given by 
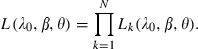
(2)

We maximize the log-likelihood of equation (2) with respect to the baseline transition intensity parameters *λ*_0_, the regression parameters *β* and the random-effect variance *θ*. This is done by using the BFGS optimization routine (Broyden, 1970) through the command in R. This yields parameter estimates together with a numerically derived Hessian matrix at the estimate values from which asymptotic standard errors for the parameter estimates are obtained. Comprehensive details of the likelihood function are provided in Appendix A.

In the following subsections, we describe analyses that use the four-state model to investigate firstly the extent to which a symmetric disease pattern exists in the hands of psoriatic arthritis patients and then the relationship between activity and damage at the joint level.

### 3.2. Patterns—symmetric joint damage in the hands

In RA, there is a consensus among rheumatologists that damage is highly symmetric. This, however, is not so for psoriatic arthritis, where there is still uncertainty among rheumatologists concerning the extent of symmetry (Gladman, 2005, 2006). To inform this debate, at least with regard to the hands of psoriatic arthritis patients, we analyse the data from the 14 pairs of joints within each of the 517 patients by using the correlated multistate processes model that was described earlier. Table 1 presents the observed transition table for this model. We find that there were 70169 observed transitions, of which only 687 were actual transitions between differing states, over all pairs of joints in the 517 patients, with 813 hand joints becoming damaged over the follow-up period of the study out of the possible 14476 total hand joints under investigation.

**Table 1:** Observed joint transitions for the four-state multistate model

Transition from	Number of transitions to the following states:			
	
	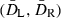	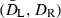	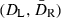	(*D*_L_,*D*_R_)
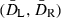	66729	225	204	126
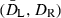	0	1339	0	81
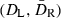	0	0	1414	51
(*D*_L_,*D*_R_)	0	0	0	0

To investigate the extent of disease symmetry in the hands we set the explanatory vector 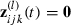 in equation (1) and reparameterize some of the baseline transition intensities in terms of others, i.e. we specify 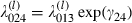 and 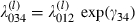. Therefore a test for symmetry would require 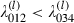 and 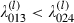, or equivalently that *γ*_24_>0 and *γ*_34_>0, i.e. a symmetrical damage pattern implies that the tendency for a joint at a specific location to become damaged is increased if the contralateral joint on the other hand is earlier damaged, and this applies at all joint locations. Note that the usage of symmetry here is a rheumatological usage and does not refer to the technical use of the term symmetry in statistics to describe the invariance of cell probabilities in the permutation of the subscripts indexing these values, as when applied to describing some statistical models based on repeated categorical response data.

Table 2 presents the results from fitting the model to investigate symmetry. The parameters *γ*_24_ and *γ*_34_ are estimated to be 1.82 and 1.39 respectively with corresponding 95% confidence intervals of (1.49,2.14) and (1.01,1.78). Both of these confidence intervals indicate substantial departures from zero to the right and therefore present strong evidence for symmetry. This symmetry, in turn, implies that there are local dependences in both directions between the two damage subprocesses (i.e. the left and the right) of the composable multistate process.

**Table 2:** Parameter estimates together with associated 95% confidence intervals (in parentheses) for the model fitted to investigate symmetry in the left and right damage processes†

*Joint*	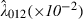	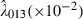
MCP1	0.91 (0.65, 1.29)	0.34 (0.20, 0.57)
MCP2	0.31 (0.19, 0.52)	0.17 (0.08, 0.33)
MCP3	0.20 (0.11, 0.37)	0.36 (0.22, 0.59)
MCP4	0.62 (0.41, 0.94)	0.63 (0.42, 0.95)
MCP5	0.35 (0.21, 0.58)	0.68 (0.46, 1.02)
PIP1	0.95 (0.65, 1.38)	0.78 (0.53, 1.15)
PIP2	0.66 (0.44, 0.99)	1.16 (0.82, 1.64)
PIP3	0.67 (0.47, 0.97)	0.30 (0.19, 0.49)
PIP4	0.34 (0.21, 0.56)	0.20 (0.11, 0.37)
PIP5	0.20 (0.11, 0.37)	0.37 (0.23, 0.59)
DIP2	0.50 (0.33, 0.77)	0.57 (0.37, 0.87)
DIP3	0.41 (0.26, 0.64)	0.36 (0.23, 0.56)
DIP4	0.96 (0.66, 1.39)	0.82 (0.55, 1.22)
DIP5	0.74 (0.50, 1.10)	0.42 (0.27, 0.65)

† The estimate for the random-effect variance is 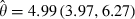. Estimates for the symmetry parameters are 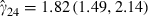 and 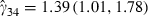. MCP, metacarpophalangeal; PIP, proximal interphalangeal; DIP, distal interphalangeal.

We now consider whether the relationship between disease activity and damage, which is repeatedly found at the patient level, is seen at the individual joint level.

### 3.3. Regression of activity on damage at joint level

To investigate the relationship between damage and the dynamic course of disease activity at the individual joint (location) level we use the model represented by equation (1) with the explanatory variable vector process **z**(*t*) defined by information on activity. We define binary indicators of joint level activity, *A*_L_(*t*) and *A*_R_(*t*) for the left and right joints respectively in a pair at time *t*. In these variables, we make no distinction between activity in the form of tenderness and effusion. However, for transitions into a state of damage, we are interested in assessing a possible dose–response relationship between joint activity and the rate at which damage occurs. Thus we also define dynamic binary indicators for joint level tenderness only, *T*_L_(*t*) and *T*_R_(*t*), and joint level effusion (with or without tenderness but usually tender), *E*_L_(*t*) and *E*_R_(*t*), for the left (L) and right (R) hands at time *t*. Hence, assuming that no previous damage has occurred to either joint (state 1), models for the transition intensities out of state 1 are given by 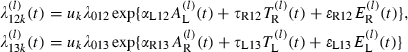
(3) where *β*_12_=(*α*_L12_,*τ*_R12_,*ɛ*_R12_)^T^, *β*_13_=(*α*_R13_,*τ*_L13_,*ɛ*_L13_)^T^ and we assume that the baseline transition intensities are constrained to be the same across the 14 hand joint locations.

When forming models for the transition intensities into state 4, we choose not to include information (in the form of explanatory variables) on the activity process in the opposite (damaged) joint at time *t* because of the dominant effect of symmetrical damage. Hence, our models for the transition intensities into state 4 are given by 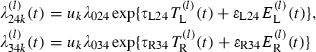
(4) with *β*_24_=(*τ*_L24_,*ɛ*_L24_)^T^, *β*_34_=(*τ*_R34_,*ɛ*_R34_)^T^ and, once again, we assume that the baseline transition intensities are constrained to be the same across the 14 joint locations.

Because of the panel nature of the data, we focus on the relationship between observed transitions in the damage process and the values of these activity variables (which are represented by activity or joint pain and swelling on both the left and the right hands) at the *last* clinic visit, i.e. we assume that *A*_L_(*t*), *A*_R_(*t*), *T*_L_(*t*), *T*_R_(*t*), *E*_L_(*t*) and *E*_R_(*t*) are piecewise constant between clinic visits. Thus underlying activity is assumed to be coarsened by the timing of clinic visits. We additionally assume that these activity variables can only change ‘state’ immediately after the time of a visit to the clinic when damage information becomes available.

It is ‘biologically’ conceivable that this model, given by expressions (3) and (4), can be further constrained to allow *α*_L12_=*α*_R13_, *τ*_R12_=*τ*_L13_, *τ*_L24_=*τ*_R34_ and, similarly, *ɛ*_R12_=*ɛ*_L13_ and *ɛ*_L24_=*ɛ*_R34_. We applied these constraints to subsequent models in the interest of parsimony.

However, the analysis that was just described did not fully exploit the dynamic potential of the framework being used, as it only used the current visit activity information when predicting future damage. It is quite plausible that some measure(s) of the ‘activeness’ of the joint over time may better characterize the relationship between activity and damage at the joint level, since more of the history of the disease activity process can be incorporated. For illustration, in a further analysis, we added binary explanatory variables (for joints on the left and right hands) that now indicate whether a joint was ever observed active (either swollen or tender) at the present visit or any of the previous protocol visits. We included similar constraints on the explanatory variables (i.e. *ρ*_L12_=*ρ*_R13_, *ρ*_R12_=*ρ*_L13_ and *ρ*_L24_=*ρ*_R34_, where *ρ* is the coefficient corresponding to whether or not the joint has ever been active) and allowed only prior activity in the undamaged joint to have an effect on the corresponding transition into state 4 (i.e. *ρ*_L34_=*ρ*_R24_=0). We considered the possibility of an effect of activity at other joints on the damage rates by including binary explanatory variables to indicate whether or not activity occurred at any other joint in the same hand and whether or not activity occurred at any other joint in the opposite hand. The addition of these explanatory variables produced no demonstrable effect and they were subsequently removed from the model. Furthermore, we also fitted this model including a four-level factor to account for joint type (levels: finger metacarpophalangeal, proximal interphalangeal and distal interphalangeal and thumb metacarpophalangeal) with each level of this variable constrained to have the same effect across all transitions. However, this was found not significantly to affect any of the transitions to damage and was subsequently removed from the model. Some previous analyses (e.g. Bond *et al.* (2007)) have suggested that the erythrocyte sedimentation rate is one of the most likely patient confounders for a relationship between activity and damage in the hand joints. Thus, we fitted a model that included an explanatory variable to represent time varying erythrocyte sedimentation rate, constrained to have the same effect across all transitions. The inclusion of this explanatory variable did not change substantially the estimated effects of the activity explanatory variables on the transitions to damage, and hence the time varying erythrocyte sedimentation rate variable, for which there was also no evidence for an effect in this model, was not included in our final model.

Table 2 shows variation in baseline transition intensities fitted to each joint location but no obvious patterns. We considered the fitting of joint-location-specific transition intensities in our final model. However, such a model would require a large number of extra parameters to be estimated together with their associated standard errors. This would be challenging computationally and the relatively low number of transitions from states 2 and 3 to state 4 (shown in Table 1) may result in unstable parameter estimates. We take the view that some assumptions must be made with regard to parameter constraints in the interests of parsimony. The inability to model differences in the baseline rates among the joint locations easily and the need to maintain sufficient parsimony are practical limitations of this model.

Tables 3 and 4 present the results of fitting the constrained model including previous activity to 510 of 517 patients included in the earlier ‘symmetry’ analysis. Seven patients were excluded owing to missing information on disease activity. In Table 3‘transitive’ joint refers to the joint undergoing the transition to a state of damage (i.e. to state 2, 3 or 4) and ‘opposite’ joint refers to the same joint in the opposite hand. From Table 3, where there is no previous damage in either joint (i.e. the model is currently in state 1), we observe increases in the rates of transitions into a state of damage when there is current activity (in the form of both tenderness and effusion) and when there has been some past activity in the transitive joint, compared with when no activity has been seen. We note that effusion shows a larger positive effect on transition to damage than tenderness. Conversely, activity in the opposite joint appears not to affect the transition rate to damage significantly. Where the opposite joint is already damaged we see an increase in the transition to damage both where there is current tenderness and where there is current effusion as well as where past activity has occurred in the transitive joint. We note that the effects of tenderness and effusion are similar; we no longer see an apparent dose–response relationship for the covariates representing activity in the transitive joint. This perhaps indicates that, when the opposite joint becomes damaged, the damage process in a joint is more complicated than when no such opposite damage exists and may be significantly influenced by other biological processes.

**Table 3:** Log-intensity ratio and intensity ratio estimates for activity at the individual joint level, together with associated 95% confidence intervals (in parentheses)

Effect on transition to damage	Estimate	Intensity ratio
*No previous damage in either joint*		
Tenderness in the transitive joint	1.01 (0.72, 1.31)	2.76 (2.06, 3.70)
Effusion in the transitive joint	1.50 (1.22, 1.77)	4.47 (3.38, 5.90)
Activity in the opposite joint	0.17 (−0.10, 0.44)	1.18 (0.90, 1.55)
Transitive joint active in the past	0.76 (0.52, 1.00)	2.14 (1.68, 2.71)
Opposite joint active in the past	0.10 (−0.15, 0.35)	1.10 (0.86, 1.41)
*Opposite joint damaged*		
Tenderness in the transitive joint	0.81 (0.41, 1.20)	2.24 (1.51, 3.32)
Effusion in the transitive joint	0.78 (0.34, 1.23)	2.19 (1.40, 3.41)
Transitive joint active in the past	0.31 (0.01, 0.62)	1.37 (1.00, 1.86)

**Table 4:** Baseline intensities *λ* and random-effect variance parameter *θ*, together with associated 95% confidence intervals (in parentheses)

*Parameter*	*Estimate (*×10^−2^*)*	*95% confidence interval (*×10^−2^*)*
*λ*_012_	0.28	(0.21, 0.36)
*λ*_013_	0.27	(0.21, 0.34)
*λ*_024_	2.15	(1.49, 3.10)
*λ*_034_	2.34	(1.58, 3.47)
*θ*	3.81	(2.98, 4.88)

The log-intensity ratios of the six transitive association effects that were described in the previous paragraph are all positive and large, with probable differential effects observed between the comparable transitions for tender-only and the more severe effused (usually also tender) joints, ipsilaterally, where no damage has occurred to the opposite joint. The effects corresponding to effusion tend to be larger than the comparable effects for tender-only joints, ipsilaterally, again where no damage has occurred to the opposite joint. These six association effects are all the statistically significant effects that were found and the four corresponding to current activity suggest that the link between activity and damage is local or specific to the joint on the particular hand being considered. These four associations are what we would consider as a local dependence or influence of activity on damage at a joint. We do not observe any statistically significant association, or evidence of a possibly substantive effect, of having or not having activity in a joint on one hand with having or not having damage on the contralateral joint of the other hand.

All the analyses that are reported in this subsection treated the disease activity process as explanatory variables and the damage process as the outcome. In addition, the various activity explanatory variables were treated as remaining constant between clinic visits. Such models are extremely important because the explanatory variables represent the information that is available to clinicians in managing a patient's disease. However, if the aim is to understand the relationship between activity and damage at a more fundamental level, then it is important to recognize that the disease activity process can be a highly variable and dynamic process (especially when medication is available to treat disease activity). Therefore, with intermittently measured activity (as well as damage) and possibly highly variable gap times between clinic visits, the assumption of piecewise constancy of explanatory variables may be unsuitable for some purposes.

In the next section, we address this concern by considering a multistate model which jointly defines the activity and damage processes (i.e. both activity and damage are treated equally as outcomes).

## 4. Three-state model for activity–damage process

To characterize better the temporal relationship between activity and damage, we propose a new three-state model for each of the 28 phalangeal joints across the left and right hands of a psoriatic arthritis patient. The three states of this model, which combines activity and damage events, are defined as

- state 1, no damage 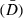 and no activity 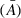 in the joint,
- state 2, no damage 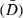 and activity (*A*) in the joint, and
- state 3, damage (*D*) in the joint.

**Fig. 3:**
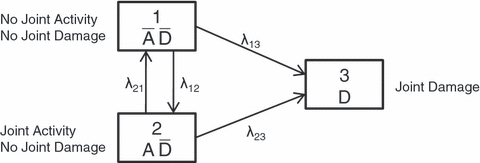
Diagram of the multistate model for the activity and damage combined process

A diagrammatic representation of this model is shown in Fig. 3. Transitions between states 1 and 2 (i.e. between the ‘activity’ states) are permitted. The damage state (i.e. state 3) is absorbing and therefore follow-up for a specific joint ends when damage occurs. Thus, observation of the multistate process for a joint is stopped either when damage occurs or at the last visit to the clinic.

Correlation between the 28 multistate joint processes within the hands of a psoriatic arthritis patient is expected and this within-patient correlation needs to be incorporated in the model. However, because of the reversibility of transitions between activity states, it is computationally difficult to introduce subject-specific random effects into this model as done for the four-state model of Section 4. Whereas Cook *et al.* (2004) achieved this with a simple discrete distribution for the random effects in the progressive multistate model setting, we instead adopt a ‘generalized estimating equation’ approach to estimation, in which we assume a working independence correlation structure for the 28 correlated multistate hand joint processes within a patient, and then calculate robust standard errors for the estimated parameters through use of the robust information sandwich matrix (Cox, 1961; Huber, 1967; White, 1982; Royall, 1986). Our approach is similar to that employed in Lee and Kim (1998) but now for non-progressive clustered processes.

The analysis is performed by using the package (Jackson, 2008) in R, which implements a standard maximum likelihood estimation approach for multistate models with panel data (Kalbfleisch and Lawless, 1985). This package allows the user to specify explanatory variable effects to act on certain transition intensities only and yields maximum likelihood estimates for the baseline transition intensities and explanatory variable effects. We extract a score vector for each patient, calculated at the maximum likelihood estimates, together with the numerically derived Hessian matrix from the optimization in the package to obtain an asymptotic robust variance–covariance matrix for the maximum likelihood estimates, using a method presented in Cook and Lawless (2007) (page 342). This robust variance–covariance matrix accounts for the correlation between joints without the use of a subject-specific random effect.

More precisely, we consider for the *k*th patient at the *p*th phalangeal joint of the hands (*p*=1,…,28), a multistate process 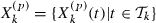, with state space Ω={1,2,3} as described above, and 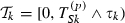 denoting the time interval of interest (measured in years) which begins at entry to the clinic and stops at the visit to the clinic where damage in the *p*th phalangeal joint is first observed, if it occurs, or else stops at the last visit to the clinic. Let 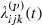 be the transition intensity function associated with 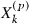 corresponding to the transition *i*→*j*, where *i*≠*j* (*i*=1 or *i*=2;*j*=1,2,3). Let 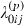 be the baseline transition intensity corresponding to this *i*→*j* transition. In addition, let 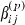 be a vector of regression parameters associated with the predictable explanatory variable vector process 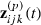. Then the transition intensity function for the *i*→*j* transition, *i*≠*j*, at the *p*th phalangeal joint of the hands for the *k*th patient is given by 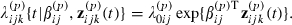
(5) For parsimony and because we believe that these 28 multistate activity–damage processes describe a similar phenomenon, we assume that 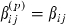 and that 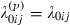 in equation (5).

The analysis of the individual joint level data which uses this three-state model is described in the next subsection. The analysis is based on the 510 patients with complete joint level information on activity and damage.

### 4.1. Jointly modelling activity and damage at joint level

The observed joint transition table for the three-state model that is illustrated in Fig. 3 is shown in Table 5. There are 134295 observed joint transitions among all the hand joints from the 510 patients. This corresponds to approximately nine transitions observed per patient hand joint. The actual number of observed transitions between differing states was 14856. By the end of the study period 772 hand joints were damaged, which corresponds to 5% of all 14280 (=28×510) hand joints. The total hand-joint-years of follow-up until damage or administrative censoring was 122008.6 years, and the observed rate of hand joint damage is six joints per 1000 hand-joint-years of follow-up.

**Table 5:** Observed joint transitions for the three-state activity–damage multistate model

*Transition from*	*Number of transitions to the following states:*		
	
	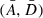	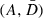	*D*
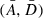	113793	6765	510
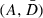	7319	5646	262
*D*	0	0	0

We fit to these data the three-state model defined in equation (5), with a five-level factor variable for type of joint (i.e. metacarpophalangeal, proximal interphalangeal, distal interphalangeal, metacarpophalangeal 1 and proximal interphalangeal 1—see Fig. 1), a time-dependent explanatory variable indicating whether the joint at the previous visit to the clinic had ever been observed active (at this visit or in the past) and a time-dependent variable indicating whether or not the corresponding joint in the opposite hand is in a state of damage for **z**(*t*). We fitted this model with explanatory variable effects on all transitions and performed Wald tests, using the robust variance–covariance matrix, to determine whether or not effects on all transitions were required. As a result, the time-dependent explanatory variable representing past activity and the joint type factor were excluded from the equations for the transition intensities out of state 2. Additionally, we parameterized the baseline 2→3 transition intensity in terms of the baseline 1→3 transition intensity, i.e. *λ*_023_=*λ*_013_ exp (*γ*), adjusting for explanatory variables.

Table 6 shows the results of fitting this model. We observe strong evidence to support the hypothesis that *λ*_023_>*λ*_013_. This evidence is reflected in a large *γ*-estimate of 3.08 (robust 95% confidence interval (1.76, 4.40)), which equates to an approximately 22 times greater transition rate to damage from an active (but not damaged) state than from a state of non-activity (and not damaged) just before the transition to damage, other factors being the same. In addition, there is evidence of the ‘ever active’ variable being positively associated with the 1→2 and 1→3 transitions. Thus a joint ever being recorded as active before the current visit increases both the baseline transition rates of making an instantaneous transition from a state of ‘no damage, no activity’ to ‘no damage, activity’ and to ‘damage’. In controlling for damage in the same joint of the opposite hand, we see that damage on the opposite hand has a significant effect only on the transitions to damage (i.e. 1→3 and 2→3), further strengthening the argument for symmetric damage seen in the previous four-state model.

**Table 6:** Intensity ratio and baseline intensity parameter estimates together with associated naive and robust 95% confidence intervals for the model that includes the effects of joint type, previous activity on transitions from state 1 and opposite damage on all transitions

Parameter	Estimate	95% confidence interval	
		
		*Naive*	*Robust*
*Baseline intensities(*×10^−2^*)*			
*λ*_012_	9.24	(8.19, 10.43)	(6.76, 12.63)
*λ*_013_	0.14	(0.07, 0.30)	(0.04, 0.49)
*λ*_021_	151.48	(146.10, 157.10)	(126.25, 181.76)
*Intensity ratios*			
exp(*γ*)	21.72	(11.89, 39.69)	(5.79, 81.46)
Metacarpophalangeal on 1→2	1.10	(0.99, 1.21)	(0.82, 1.47)
Metacarpophalangeal on 1→3	0.37	(0.16, 0.86)	(0.05, 2.75)
Proximal on 1→2	1.24	(1.12, 1.36)	(0.96, 1.59)
Proximal on 1→3	1.04	(0.56, 1.94)	(0.31, 3.52)
Distal on 1→2	0.86	(0.77, 0.95)	(0.63, 1.16)
Distal on 1→3	2.82	(1.60, 4.96)	(0.95, 8.36)
Thumb metacarpophalangeal on 1→2	1.26	(1.12, 1.42)	(0.97, 1.63)
Thumb metacarpophalangeal on 1→3	2.68	(1.43, 5.04)	(0.88, 8.13)
Joint ever active on 1→2	2.98	(2.84, 3.13)	(2.13, 4.17)
Joint ever active on 1→3	3.07	(2.38, 3.96)	(1.27, 7.40)
Opposite joint damage on 1→2	1.20	(1.01, 1.44)	(0.64, 2.27)
Opposite joint damage on 1→3	7.19	(4.90, 10.54)	(2.11, 24.44)
Opposite joint damage on 2→1	1.08	(0.91, 1.28)	(0.64, 1.82)
Opposite joint damage on 2→3	7.50	(5.08, 11.09)	(2.40, 23.46)

The value exp(*γ*)=21.72 describes the ratio between *λ*_23_ and *λ*_13_ in the absence of damage to the opposite joint. Since the effects of damage to the opposite joint are of similar magnitude, we obtain a similar estimate of 21.72×7.50/7.19=22.65 for this ratio in the presence of damage to the opposite joint. This concurs to some extent with the results from the four-state model which showed that the presence of activity has a significant effect on the rate of transition to damage in a joint where the same joint in the opposite hand already exhibits damage. Furthermore, we note that there is a negligible effect of opposite joint damage on the 1→2 transition intensity. This suggests that the apparent symmetric effect of opposite joint damage is not mediated through an increase in disease activity but rather through another, unknown, mechanism.

Further generalization of our three-state model to incorporate piecewise constant baseline intensities resulted in some evidence for a decline in the transition rate from the state of ‘no damage, no activity’ (state 1) to the state of ‘no damage, activity’ (state 2) as the time in clinic increased. However, no evidence was found for changes over time in the transition rate from state 2 to state 1 and the two transition rates into the ‘damage’ state (state 3). Moreover the *γ*-estimates that were obtained when the follow-up time period was stratified into three intervals either as [0,1), [1,3) and [3,∞) or [0,5), [5,10) and [10,∞) were 3.155, 3.015 and 2.714 or 3.048, 2.618 and 2.807 respectively. These estimates were well within the 95% robust confidence interval of 1.76–4.40 that was reported in the previous paragraph and provided no evidence for a marked change in *γ* over time. Finally, there was no demonstrable modification of the estimated explanatory variable effects in Table 6 when these additional time inhomogeneous three-state models were fitted.

The analyses of this subsection again demonstrate a local dependence at the joint level between activity and damage (i.e. activity influences damage locally) and characterize more fully the temporal ordering.

## 5. Causality

### 5.1. Framework

In the section on ‘Assessing causality’ in a classic epidemiological text (Lilienfeld and Stolley, 1994) it is argued that

‘a relationship is considered causal whenever evidence indicates that the factors form part of the complex of circumstances which increases the probability of the occurrence of disease and that a diminution of one or more of these factors decreases the frequency of the disease’.

Sir Austin Bradford Hill, in his 1965 Presidential address to the Section of Occupational Medicine of the Royal Society of Medicine (Hill, 1965), discussed aspects of an association that should be especially considered when attempting to infer causation in this sense from association. These aspects, which are now referred to as the Bradford Hill criteria, are

- strength of association,
- consistency,
- specificity,
- temporality,
- biological gradient,
- biological plausibility,
- coherence,
- experimental evidence (when available) and
- analogy.

However, Hill never intended these ‘viewpoints’ to be ‘hard-and-fast rules of evidence that *must* be obeyed before we can accept cause and effect’ (Hill, 1965). They are not necessary and/or sufficient conditions to declare causation from an observed association, although temporality is indisputably a necessary condition as a cause must precede its effect. They are, however, useful in providing a structure when attempting to move from association to causation. In particular, they (excluding criterion (h)) have been found to be valuable in epidemiological settings.

Schweder (1970) regarded his concept of local (in)dependence as a potential aid in addressing causal questions and it is closely linked to the concept of Granger causality (Granger, 1969). We note that Granger causality was initially defined in the context of discrete time series, where suppose that there are two time series *X*(*t*) and *Y*(*t*), and 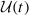 represents ‘all of the information in the universe’ up to time 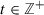. Then we say that *X*(*t*) is Granger causal for *Y*(*t*) if *Y* may be better predicted at time *t*+1 given 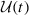 than given 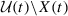. Otherwise we say that *X*(*t*) is Granger non-causal for *Y*(*t*). Local (in)dependence may be viewed somewhat as an extension of the Granger causality–non-causality concept to processes in continuous time. The asymmetry of the local independence concept makes it particularly attractive, as having one subprocess locally influencing a change in another, but the other not having any influence on the first, is precisely how we would like to characterize a causal effect of *Y*_*j*_ on *Y*_*k*_. However, a one-sided local dependence relationship between two subprocesses of a composable Markov process is not sufficient to imply causation.

Aalen (1987), in extending the concept, stressed that local (in)dependence was a dynamic statistical approach which, by incorporating time explicitly, offers a natural way to model potential causal relationships. This is reflected in other writings which take a dynamic viewpoint including that of Arjas and Parner (2004) who not only believe that explicit accounting of the time aspect should be made because of the time ordering of cause and effect but also because often the durations between different events are an integral part of the causal problem and therefore of the analysis. This viewpoint is also seen in Commenges and Gégout-Petit (2009) who developed a general dynamical model as a framework for causal interpretation, which uses the concepts of ‘the system’ and ‘causal influence’, is centred on stochastic processes and further builds on the local dependence concept.

In our discussion of psoriatic arthritis disease progression that follows, we use both the Bradford Hill criteria and Schweder's local (in)dependence concept to reason about whether associations that were found in our analyses, which are based on multistate models at the individual joint level of the hands, can be considered causal. We recognize that there are other formal frameworks for thinking about causality (e.g. the decision theoretic (conditioning by intervention, do-calculus), counterfactuals (potential outcomes) and causal graphs (marginal structural models) approaches) that have been applied in the literature (Dawid, 2000; Pearl, 2000; Rubin, 1974, 1978; Robins *et al.*, 2000). However, we believe that, for our application, the Bradford Hill criteria and Schweder's local (in)dependence are more natural and helpful methods for inferring causal relationships, as our focus is not on intervention but on further mechanistic understanding of the disease. See Aalen and Frigessi (2007) for a discussion of mechanistic causality.

### 5.2. Information for causal inference

As described previously, recent work (Bond *et al.*, 2007; Bond and Farewell, 2009) has demonstrated that higher prior active joint counts are related to higher rates of damage development at the patient level. Also, the differential effects of tender only and the more severe effused (usually also tender) provide some indication of a dose–response relationship. The analyses of Sections 3 and 4 allow us to consider the extent to which these analyses performed at the individual joint level, and the associations that may be demonstrated, aid our understanding of disease progression and inform us additionally about causality.

In addition to the recognition that association cannot be used to prove causation, inference about causation also needs to reflect the possibility of different causal pathways. Thus, a link between activity and damage at the individual joint level need not preclude the involvement of other factors nor completely separate pathways to damage.

In Section 3.2, evidence emerged of a symmetric pattern of joint damage. For a pair of joints at the same location in the two hands, local dependences were identified in both directions between the damage subprocesses in the left and right hands. Although these results represent an important finding, these local dependences do not immediately warrant a causal explanation, as they do not produce a one-sided (local dependence) asymmetric relationship between the two damage subprocesses. It is quite plausible that the same underlying biological mechanism is driving these two damage subprocesses, although a robust biological explanation for damage symmetry has not been put forward and tested yet. However, there are some animal data to suggest neurological influence on inflammation (Chahl and Ladd, 1976; Denko and Petricevic, 1978), which, as Levine *et al.* (1987), Helliwell *et al.* (2000) and Bukhari *et al.* (2002) have discussed, may indicate that

‘a biological mechanism exists whereby afferent nerves from one joint can induce an inflammatory response in the contralateral joint by inducing the release of inflammatory mediators’

(Bukhari *et al.*, 2002). Although this explanation is somewhat speculative, it would account for the observed but non-causal relationship of damage in one hand being associated with subsequent damage in the other hand.

Thus, in exploring causal links between activity and damage at the individual joint level, it must be accepted that any link will be part of a complex of factors that influence the extent of damage seen in psoriatic arthritis patients.

However, the results in Section 3.3 do suggest

- joint specificity of the relationship between activity and damage;
- strong associations for the four statistically significant and biologically plausible local effects obtained and, where no previous damage has occurred in either joint of a pair,
- a dose–response relationship of activity with damage at the joint level (i.e. a biological gradient).

Similar effects of tenderness and effusion, rather than a dose–response relationship, were seen where the opposite joint of a pair exhibits damage, though we note that the damage process may become more complex once the opposite joint is damaged, especially in light of the symmetric relationships that were discussed in Section 3.2. If other causal pathways influence damage where opposite damage exists then a dose–response relationship may be more difficult to detect. As indicated in Section 3.3, we would consider these relationships to represent local dependence of activity on damage although the activity process is not formally modelled. Here and in Section 4 the determining of asymmetry of local dependence relationships is not specifically addressed and is less critical because any influence of damage on subsequent activity in a joint is of less clinical interest or, at least, represents a very different clinical question. However, as Aalen argued, the dynamic perspective of Schweder's work may be most important and the analysis of Section 3.3, because of the longitudinal nature of the data, does help to characterize the temporal relationship between activity and damage.

Therefore these results do provide support for a putative causal relationship between activity and damage. It can also be argued, we feel, that, from the perspective of both biological plausibility and temporal ordering, this demonstrated relationship at the joint level offers more support for a causal link than the relationships at the patient level that were seen in previous investigations.

Results from the three-state model in Section 4 confirm that the link between activity and damage at the individual joint level is seen even in a model which more realistically reflects the highly variable nature of the activity process. This further strengthens the evidence for the association and the temporal ordering and, thus, for a causal link. Moreover, although there is some increase in the baseline transition rate from the inactive state if the joint has ever been active, from 0.0014 to 0.0014×3.07=0.0043, this is much less than the rate from the active state, 0.0304. Thus, the causal argument is enhanced since the disappearance of activity has a similar (but negative) effect to that of the appearance of activity. We note also that in this model, where activity is modelled in continuous time, there is no evidence to suggest that the presence of damage in the opposite joint of a pair diminishes the increase in the transition rate to damage from a state of activity when compared with that from a state of inactivity.

### 5.3. Does activity cause damage?

The determination of causality from observational data is notoriously difficult. Mathematical formalization of the causality concept, in itself, is not enough to allow a causal relationship to be inferred from an observed association. However, the presence of a mathematical framework to reason about causality is helpful, if not essential, when attempting to address clinical questions of a causal nature. The multistate modelling framework provides a good illustration of how mathematical and statistical concepts, such as local independence and local dependence, allow causal hypotheses to be framed in a way such that they can be investigated in a statistically coherent manner. Mathematical formalization together with sound application of the Bradford Hill criteria may represent a useful strategy for inferring causality from observational data.

Because causation cannot be proved with observational data, there can be debate about the extent to which causality is supported. For example, activity might be regarded as an early stage of a damage process so that their temporal ordering is simply a natural course of disease with no causal implications. However, because activity is clearly part of an inflammatory process which is generally transient and damage is a condition which is irreversible and need not be associated with concurrent inflammation, some sort of causal link appears more reasonable. This argument is more reasonable because we have been able to demonstrate, to a considerable degree, that this damage process will occur at the joint level. Of course, if we did not find this joint level relationship, there might be other causal explanations for previous findings relating patient level associations between the extent of activity and progression of damage. These arguments must, however, be somewhat more complex and speculative and that is why we would argue that the establishment of an activity–damage relationship at the joint level ‘strengthens’ the case for a causal pathway.

Similarly, investigation of the biological gradient, or dose–response, Bradford Hill criterion can be context specific in its application. For example, most diets in the developed world contain sufficiently high levels of phenylalanine to induce neurological manifestations for individuals lacking the enzyme phenylalanine hydroxylase. However, elsewhere, a dose–response relationship will exist between the level of phenylalanine and the level of neurological problems. Thus the primary risk factor for the disease phenylketonuria is binary (genetic) when the gene is rare and a continuous dietary measure where the genetic condition is common.

One might therefore consider a ‘trigger’ hypothesis for the relationship between activity and damage. If the onset of inflammation is all that is needed to initiate a process leading to damage, then no dose–response or more complex temporal relationship with activity over time would be seen. However, conversely, if we do see such relationships then such a ‘trigger’ hypothesis is less plausible.

Previous analyses (see Bond *et al.* (2007) for a summary and further references) suggested erythrocyte sedimentation rate as the most likely patient level confounder of activity and damage relationship, and it was included in those analyses. As reported in Section 3.3, it did not have a demonstrable effect in models at the joint level. The use of random effects will mitigate the potential influence of other unmeasured confounders at the patient level. Potential confounders measured at the individual joint level are not easily envisaged on the basis of published literature on arthritic damage in primarily autoimmune arthritides.

Bond and Farewell (2009) also addressed the issue of potentially informative observation times for these data. Although some evidence for such informative observation was present, allowance for it in the study did not demonstrate any marked bias in the relationship between activity and damage.

Thus the work that is reported here represents our effort to substantiate the well-held, but yet unproven, claim that activity causes damage in psoriatic arthritis. Our systematic approach to assessing this claim, which crucially relied on the analyses of activity and damage data at the hand joint level and not only at the patient level, leads us to conclude that there is strong epidemiological evidence for a causal relationship between activity and damage, although other causal pathways may also be present. This conclusion is based on our results being consistent with the majority of the Bradford Hill criteria: specificity, strength of association, biological gradient, temporality and biological plausibility, at the joint level. Others such as consistency and analogy have been shown at the patient (systemic) level in other psoriatic arthritis populations and in RA populations respectively. Moreover our results do not, in any way that we know of, conflict with generally known facts regarding the biology and natural history of progression of disease in psoriatic arthritis patients, thus suggesting coherence. Furthermore, there are recent clinical trials that have shown that new biological therapies are effective in slowing the progression of arthritis disease. These biologics calm the inflammation of arthritis by inhibiting the components of the immune system that trigger the inflammatory response.

## 6. Conclusion

The study of the progression of psoriatic arthritis by using multistate models has provided an intuitive way of examining the disease process from a dynamic perspective. We considered the damage process in the individual joints as a continuous Markov process and this was modelled by using various multistate models over a series of discrete states. In our four-state model (Section 3), we considered the transition intensities relating to the damage process to be functions of the activity process at the previous visit to the clinic in each pair of hand joints. This allowed an assessment of the influence of disease activity on joint damage, while incorporating explicitly the passage of time and the inherent correlation between the joints through the use of a subject-specific random effect. Explanatory variables that are found to affect the transitions to states of damage significantly may imply a local dependence between activity and damage, in accordance with the work of Schweder. The three-state model (Section 4) allowed joint modelling of activity and damage as continuous Markov processes and provided a further insight into how the activity process may influence the damage process. The fit of this model suggested strong evidence of an effect of disease activity on joint damage, again demonstrating a local dependence between activity and damage at the individual joint level, while accounting more appropriately for the dynamic nature of both the activity and damage processes.

Overall, the use of multistate models has proved indispensable in the assessment of local (in)dependences in dynamic processes. Our application of these ideas at the joint level in psoriatic arthritis patients has allowed us to determine the extent to which evidence of local dependence between the activity and damage processes, together with the Bradford Hill criteria, permits a causal link between activity and damage. We conclude, after consideration of other possible explanations, that there is significant evidence in our analyses to suggest a causal relationship between disease activity and clinical damage in the hand joints of patients suffering from psoriatic arthritis, although the biological mechanism by which this occurs is still to be fully understood.

Our particular attempt to move from association to causation by using Schweder's local independence and local dependence concepts and the Bradford Hill criteria is, to our knowledge, one of the first comprehensive attempts along these lines at establishing causality in a well-motivated, important and substantial medical problem. Our analyses used data from the largest cohort of psoriatic arthritis patient followed up over time in the world, with data at the most appropriate level (i.e. the joint level) for investigating the causal relationship between activity and disease progression. We recommend that this approach to inferring causality from cohort data be considered more often in the future.

## Appendix A: Likelihood derivation for four-state model

To derive the likelihood we take advantage of the fact that our model, conditional on the random effects, is Markov. Therefore as described earlier, the contribution to the conditional likelihood, *L*_*k*_(*λ*_0_,*β*,*θ*|*u*_*k*_), from the *k*th patient can be written as the double product of the transition probabilities, conditional on *u*_*k*_, over the observed trajectories, {(*x*_1*lk*_,…,*x*_*n*_*k*_*lk*_):*l*=1,…,14}, followed by the joint pairs at the 14 hand joint locations of this patient through the *n*_*k*_ visits to the clinic, i.e.

where the notation used is as in Section 3.

As we include only those patients who entered the psoriatic arthritis clinic in state 1 for all hand joint locations, each joint pair of every patient considered in our analysis undergoes one of a possible six observed progression paths throughout the study. These are as follows.

- The joint pair remains in state 1 throughout the study.
- The joint pair moves from state 1 to state 2 and remains in state 2 for the remainder of the study.
- The joint pair moves from state 1 to state 3 and remains in state 3 for the remainder of the study.
- The joint pair moves from state 1 to state 4 with the intermediate state, either 2 or 3, unknown.
- The joint pair moves from state 1 to state 2 to state 4.
- The joint pair moves from state 1 to state 3 to state 4.

Each of these possible progression paths yields a different contribution to the likelihood for each patient and, because we have a relatively straightforward progressive conditional Markov model, these transition probabilities, conditional on the subject-specific random effect, may be determined analytically.

Suppose that we have *N* patients under observation and we define the state *i*→ state *j* transition probability for joint pair *l* of the *k*th patient, conditional on *U*_*k*_=*u*_*k*_, to be

for generic times *t* and *s*, with *t*>*s*. Additionally, we define the number of study observations for the *l*th joint pair of the *k*th patient to be  of which  occur in state *i* ∈ Ω={1,…,4}, and hence . Next we let the  ordered observation times corresponding to when joint pair *l* of patient *k* is in state *i* be denoted by .

Now, assuming that the transition intensities, conditional on the random effects, are as given in model (1); then the transition probabilities  in our four-state model will be as shown in Appendix A.1. And the conditional likelihood contributions under the six possible observed progression paths for the *l*th joint pair process of the *k*th patient are as follows.

- *Progression path 1*→1:
- *Progression path1*→2:
- *Progression path1*→3:
- *Progression path1*→4:
- *Progression path1*→2→4:
- *Progression path1*→3→4:

Therefore the contribution to the conditional likelihood from joint pair *l* of the *k*th patient can be written in full generality as

where  is the indicator function taking the value 1 if joint *j* undergoes the transition progression path TP and 0 otherwise. Thus the overall contribution to the conditional likelihood from the *k*th patient may be written as

We account for all possible values of the random effect *U*_*k*_ by integrating out this term (numerically by using R). Thus the contribution to this ‘marginal’ likelihood from the *k*th patient is given by

where *U*_*k*_∼gamma(*a*,*b*), with *a*=1/*θ* and *b*=1/*θ*, i.e.

(6)

Hence the likelihood function across all *N* patients in the study is given by

(7)

We maximize function (7) with respect to the baseline intensity parameters *λ*_0_, the regression parameters *β* and the variance of the random effects, *θ*. This is done by using the command in R, which yields parameter estimates along with a numerically derived Hessian matrix at the estimate values from which asymptotic standard errors for these estimates may be obtained.

### A.1. Form of the transition probabilities

Suppose that a patient is observed at successive observation times *s* and *t* where *s*<*t*. We provide below the analytical form of the transition probabilities *p*_*ijk*_(*s*,*t*|**z**_*k*_,*u*_*k*_). For convenience, we have dropped the *l*-superscript in what follows:

Here

and we note that *β*_*ij*_ is the subvector of *β* that acts on the transition intensities from state *i* to state *j*, i.e. .

## References

[b1] Aalen OO (1987). Dynamic modelling and causality. Scand. Act. J.

[b2] Aalen OO, Borgan Ø, Gjessing HK (2008). Survival and Event History Analysis: a Process Point of View.

[b3] Aalen OO, Borgan Ø, Keiding N, Thormann J (1980). Interaction between life history events: nonparametric analysis for prospective and retrospective data in the presence of censoring. Scand. J. Statist.

[b4] Aalen OO, Frigessi A (2007). What can statistics contribute to a causal understanding?. Scand. J. Statist.

[b5] Aletaha D, Funovits J, Breedveld F, Shar J, Segurando O, Smolen JS (2009). Rheumatoid arthritis joint progression in sustained remission is determined by disease activity level preceding the period of radiographic assessment. Arth. Rheum.

[b6] Andersen PK, Keiding N (2002). Multi-state models for event history analysis. Statist. Meth. Med. Res.

[b7] Arjas E, Parner J (2004). Causal reasoning from longitudinal data. Scand. J. Statist.

[b8] Bond SJ, Farewell VT (2009). Likelihood estimation for a longitudinal negative binomial regression model with missing outcomes. Appl. Statist.

[b9] Bond SJ, Farewell VT, Schentag CT, Gladman DD (2007). Predictors for radiological damage in psoriatic arthritis: results from a single centre. Ann. Rheum. Dis.

[b10] Broyden C (1970). The convergence of a class of double-rank minimisation algorithms. J. Inst. Math. Applic.

[b11] Bukhari M, Lunt M, Harrison BJ, Scott DGI, Symmons DPM, Silman AJ (2002). Erosions in inflammatory polyarthritis are symmetrical regardless of rheumatoid factor status: results from a primary care-based inception cohort of patients. Rheumatology.

[b12] Chahl LA, Ladd J (1976). Local oedema and general excitation of cutaneous sensory receptors produced by electrical stimulation of the saphenous nerve in the rat. Pain.

[b13] Commenges D (1999). Multi-state models in epidemiology. Liftim. Data Anal.

[b14] Commenges D, Gégout-Petit A (2009). A general dynamical statistical model with causal interpretation. J. R. Statist. Soc.

[b15] Cook RJ, Lawless JF (2007). The Statistical Analysis of Recurrent Events.

[b16] Cook RJ, Yi GY, Lee K-A, Gladman DD (2004). A conditional Markov model for clustered progressive multistate processes under incomplete observation. Biometrics.

[b17] Cox DR, Neyman J (1961). Tests of separate families of hypotheses. Proc. 4th Berkeley Symp. Mathematics, Probability and Statistics.

[b18] Cox DR, Lewis PAW (1972). The statistical analysis of dependencies in point processes. Stochastic Point Processes: Statistical Analysis, Theory and Applications.

[b19] Dawid AP (2000). Causal inference without counterfactuals. J. Am. Statist. Ass.

[b20] Denko CW, Petricevic M (1978). Sympathetic or reflex footpad swelling due to crystal induced inflammation in the opposite foot. Inflammation.

[b21] Didelez V (2007). Graphical models for composable finite Markov processes. Scand. J. Statist.

[b22] Didelez V (2008). Graphical models for marked point processes based on local independence. J. R. Statist. Soc.

[b23] Gladman DD, Harris ED, Budd RC, Firestein GS, Genovese MC, Sergent JS, Ruddy S, Sledge CB (2004). Psoriatic arthritis. Kelly's Textbook of Rheumatology.

[b24] Gladman DD (2005). Discussion: Clinical features epidemiology, classification criteria and quality of life in psoriasis and psoriatic arthritis. Ann. Rheum. Dis.

[b25] Gladman DD (2006). Clinical, radiological, and functional assessment in psoriatic arthritis: is it different from other inflammatory joint diseases?. Ann. Rheum. Dis.

[b26] Gladman DD, Farewell VT (1999). Progression in psoriatic arthritis: role of time varying clinical indicators. J. Rheum.

[b27] Gladman DD, Farewell VT, Nadeau C (1995). Clinical indicators of progression in psoriatic arthritis: multivariate relative risk model. J. Rheum.

[b28] Granger CWJ (1969). Investigating causal relations by econometric models and cross-spectral methods. Econometrica.

[b29] Helliwell PS, Hetthen J, Sokoll K, Green M, Marchesoni A, Lubrano E, Veale D, Emery P (2000). Joint symmetry in early and late rheumatoid and psoriatic arthritis. Arth. Rheum.

[b30] Hill AB (1965). The environment and disease: association or causation?. Proc. R. Soc. Med.

[b31] Hougaard P (1999). Multi-state models: a review. Liftim. Data Anal.

[b32] Huber PJ, LeCam L, Neyman J (1967). The behaviour of maximum likelihood estimates under nonstandard conditions. Proc. 5th Berkeley Symp. Mathematics Probability and Statistics.

[b33] Husted JA, Gladman DD, Farewell VT, Cook RJ (2001). Health-related quality of life of patients with psoriatic arthritis: a comparison with patients with rheumatoid arthritis. Arth. Care Res.

[b34] Jackson C (2008). msm: multi-state Markov and hidden Markov models in continuous time. R Package Version 0.8.1.

[b35] Kalbfleisch JD, Lawless JF (1985). The analysis of panel data under a Markov assumption. J. Am. Statist. Ass.

[b36] Kane D, Stafford L, Bresnihan B, Fitzgerald O (2003). A prospective, clinical and radiological study of early psoriatic arthritis: an early synovitis clinic experience. Rheumatology.

[b37] Lee EW, Kim MY (1998). The analysis of correlated-panel data using a continuous-time Markov model. Biometrics.

[b38] Levine JD, Goetzl EJ, Basbaum AI (1987). Contribution of the nervous system to the pathophysiology of rheumatoid arthritis and other polyarthritides. Rheum. Dis. Clin. Nth Am.

[b39] Lilienfeld DE, Stolley PD (1994). Foundations of Epidemiology.

[b40] McHugh NJ, Balachrishnan C, Jones SM (2003). Progression of peripheral joint disease in psoriatic arthritis: a 5-yr prospective study. Rheumatology.

[b41] McKenna SP, Doward LC, Whalley D, Tennant A, Emery P, Veale DJ (2004). Development of the PsAQoL: a quality of life instrument specific to psoriatic arthritis. Ann. Rheum. Dis.

[b42] Meria-Machado L, de Uña-Álvarez J, Cadarso-Suárez C, Andersen PK (2009). Multi-state models for the analysis of time-to-event data. Statist. Meth. Med. Res.

[b43] Molenaar ETH, Voskuyl AE, Dinant HJ, Bezemer PD, Boers M, Dijkmans BAC (2004). Progression of radiologic damage in patients with rheumatoid arthritis in clinical remission. Arth. Rheum.

[b44] Mulherin D, Fitzgerald O, Breshnihan B (1996). Clinical improvement and radiological deterioration in rheumatoid arthritis: evidence that the pathogenesis of synovial inflammation and articular erosion may differ. Br. J. Rheum.

[b45] Pearl J (2000). Causality: Models, Reasoning and Inference.

[b46] Queiro-Silva R, Torre-Alonso JC, Tinture-Eguren T, Lopez-Lagunas I (2003). A polyarticular onset predicts erosive and deforming disease in psoriatic arthritis. Ann. Rheum. Dis.

[b47] R Development Core Team (2009). R: a Language and Environment for Statistical Computing.

[b48] Robins JM, Hernan MA, Brumback B (2000). Marginal structural models and causal inference in epidemiology. Epidemiology.

[b49] Royall RM (1986). Model robust confidence intervals using maximum likelihood estimators. Int. Statist. Rev.

[b50] Rubin DB (1974). Estimating causal effects of treatments in randomized and nonrandomized studies. J. Educ. Psychol.

[b51] Rubin DB (1978). Bayesian inference for causal effects: the role of randomization. Ann. Statist.

[b52] Schweder T (1970). Composable Markov processes. J. Appl. Probab.

[b53] Scott DL (2004). Radiological progression in established rheumatoid arthritis. J. Rheum.

[b54] Siannis F, Farewell VT, Cook RJ, Schentag CT, Gladman DD (2006). Clinical and radiological damage in psoriatic arthritis. Ann. Rheum. Dis.

[b55] Smolen JS, Han C, van der Heijde DMFM (2009). Radiographic changes in rheumatoid arthritis patients attaining different disease activity states with methotrexate monotherapy and infliximab plus methotrexate: the impacts of remission and tumour necrosis factor blockade. Ann. Rheum. Dis.

[b56] Sokoll KB, Helliwell PS (2001). Comparison of disability and quality of life in rheumatoid and psoriatic arthritis. J. Rheum.

[b57] Weiss NS (1986). Clinical Epidemiology: the Study of the Outcome of Illness.

[b58] Welsing PMJ, Landewé RBM, van Riel PLCM, Boers M, van Gestel AM, van der Linden Swinkels S, van der Heijde DMFM (2004). The relationship between disease activity and radiologic progression in patients with rheumatoid arthritis. Arth. Rheum.

[b59] White H (1982). Maximum likelihood estimation under mis-specified models. Econometrica.

[b60] Wright V, Moll JMH, Wright V, Moll JMH (1976). Psoriatic arthritis. Seronegative Polyarthritis.

